# Human Health Risk Assessment (HHRA) for Environmental Development and Transfer of Antibiotic Resistance

**DOI:** 10.1289/ehp.1206316

**Published:** 2013-07-09

**Authors:** Nicholas J. Ashbolt, Alejandro Amézquita, Thomas Backhaus, Peter Borriello, Kristian K. Brandt, Peter Collignon, Anja Coors, Rita Finley, William H. Gaze, Thomas Heberer, John R. Lawrence, D.G. Joakim Larsson, Scott A. McEwen, James J. Ryan, Jens Schönfeld, Peter Silley, Jason R. Snape, Christel Van den Eede, Edward Topp

**Affiliations:** 1 U.S. Environmental Protection Agency, Office of Research and Development, Cincinnati, Ohio, USA; 2 Unilever-Safety and Environmental Assurance Centre, Sharnbrook, United Kingdom; 3 Department of Biological and Environmental Sciences, Gothenburg University, Göteborg, Sweden; 4 Veterinary Medicines Directorate, Addlestone, United Kingdom; 5 Department of Plant and Environmental Sciences, University of Copenhagen, Frederiksberg, Denmark; 6 The Canberra Hospital and Canberra Clinical School, Australian National University, Canberra, Australia; 7 ECT Oekotoxikologie GmbH, Flörsheim/Main, Germany; 8 Public Health Agency of Canada, Guelph, Ontario, Canada; 9 European Centre for Environment and Human Health, Exeter University Medical School, Knowledge Spa, Royal Cornwall Hospital, Truro, United Kingdom; 10 Federal Office of Consumer Protection and Food Safety, Department 3: Veterinary Drugs, Berlin, Germany; 11 Environment Canada, Saskatoon, Saskatchewan, Canada; 12 Institute for Biomedicine, The Sahlgrenska Academy, University of Gothenburg, Göteborg, Sweden; 13 Department of Population Medicine, University of Guelph, Guelph, Ontario, Canada; 14 Environment, Health and Safety, GlaxoSmithKline, Ware, United Kingdom; 15 Umweltbundesamt Federal Environment Agency, Dessau, Germany; 16 MB Consult Limited, Southampton, United Kingdom; 17 University of Bradford, Bradford, United Kingdom; 18 Brixham Environmental Laboratory, AstraZeneca, Brixham, United Kingdom; 19 Pfizer Animal Health VMRD, Zaventem, Belgium; 20 Agriculture and Agri-Food Canada, London, Ontario, Canada

## Abstract

Background: Only recently has the environment been clearly implicated in the risk of antibiotic resistance to clinical outcome, but to date there have been few documented approaches to formally assess these risks.

Objective: We examined possible approaches and sought to identify research needs to enable human health risk assessments (HHRA) that focus on the role of the environment in the failure of antibiotic treatment caused by antibiotic-resistant pathogens.

Methods: The authors participated in a workshop held 4–8 March 2012 in Québec, Canada, to define the scope and objectives of an environmental assessment of antibiotic-resistance risks to human health. We focused on key elements of environmental-resistance-development “hot spots,” exposure assessment (unrelated to food), and dose response to characterize risks that may improve antibiotic-resistance management options.

Discussion: Various novel aspects to traditional risk assessments were identified to enable an assessment of environmental antibiotic resistance. These include *a*) accounting for an added selective pressure on the environmental resistome that, over time, allows for development of antibiotic-resistant bacteria (ARB); *b*) identifying and describing rates of horizontal gene transfer (HGT) in the relevant environmental “hot spot” compartments; and *c*) modifying traditional dose–response approaches to address doses of ARB for various health outcomes and pathways.

Conclusions: We propose that environmental aspects of antibiotic-resistance development be included in the processes of any HHRA addressing ARB. Because of limited available data, a multicriteria decision analysis approach would be a useful way to undertake an HHRA of environmental antibiotic resistance that informs risk managers.

Citation: Ashbolt NJ, Amézquita A, Backhaus T, Borriello P, Brandt KK, Collignon P, Coors A, Finley R, Gaze WH, Heberer T, Lawrence JR, Larsson DG, McEwen SA, Ryan JJ, Schönfeld J, Silley P, Snape JR, Van den Eede C, Topp E. 2013. Human health risk assessment (HHRA) for environmental development and transfer of antibiotic resistance. Environ Health Perspect 121:993–1001; http://dx.doi.org/10.1289/ehp.1206316

## Introduction

**Figure 1:**
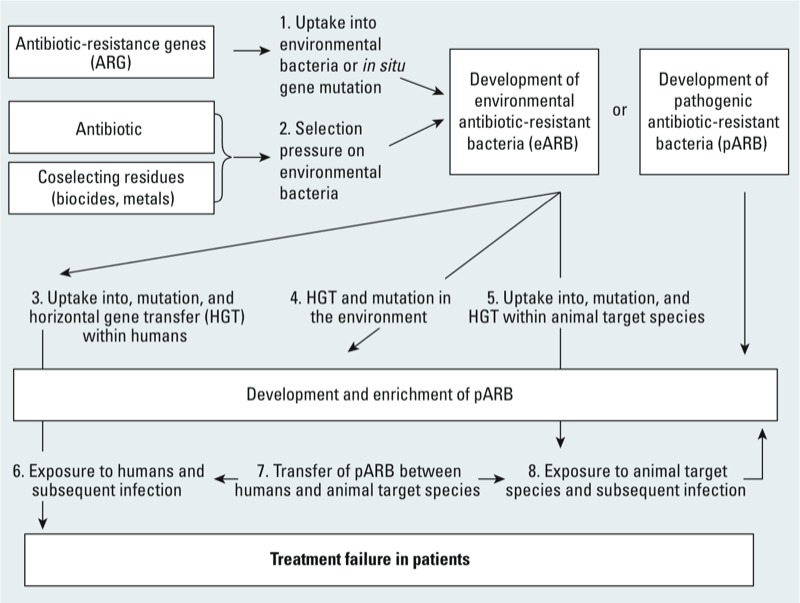
Conceptual model describing the environmental pathways that result in an increased risk of human and animal infection with antibiotic-resistant bacteria. Processes 1–6 are further described in the text. Because processes 7 and 8 are not driven by environmental factors, they are not discussed in detail.

A workshop (Antimicrobial Resistance in the Environment: Assessing and Managing Effects of Anthropogenic Activities), held in March 2012 in Québec, Canada, focused on antibiotic resistance in the environment and approaches to assessing and managing effects of anthropogenic activities. The human health concern was identified as environmentally derived antibiotic-resistant bacteria (ARB) that may adversely affect human health (e.g., reduced efficacy in clinical antibiotic use, more serious or prolonged infection) either by direct exposure of patients to antibiotic-resistant pathogen(s) or by exposure of patients to resistance determinants and subsequent horizontal gene transfer (HGT) to bacterial pathogen(s) on or within a human host, as conceptualized in Figure 1. ARB hazards develop in the environment as a result of direct uptake of antibiotic-resistant genes (ARG) via various mechanisms (e.g., mobile genetic elements such as plasmids, integrons, gene cassettes, or transposons) and/or proliferate under environmental selection caused by antibiotics and coselecting agents such as biocides, toxic metals, and nanomaterial stressors (Qiu et al. 2012; Taylor et al. 2011), or by gene mutations (Gillings and Stokes 2012). Depending on the presence of recipient bacteria, these processes generate either environmental antibiotic-resistant bacteria (eARB) or pathogens with antibiotic-resistance (pARB) (Figure 1).

Human health risk assessment (HHRA) is the process used to estimate the nature and probability of adverse health effects in humans who may be exposed to hazards in contaminated environmental media, now or in the future [U.S. Environmental Protection Agency (EPA) 2012]. In this review we focus on how to apply HHRA to the risk of infections with pathogenic ARB because they are an increasing cause of morbidity and mortality, particularly in developing regions (Grundmann et al. 2011). An antimicrobial-resistant microorganism has the ability to multiply or persist in the presence of an increased level of an antimicrobial agent compared with a susceptible counterpart of the same species. For this review, we limited the resistant group of microorganisms to bacteria and therefore to antibiotic resistance, an area in which the term “antibiotic” is used synonymously with “antibacterial.” It is important to understand the contribution that the environment has on the development of resistance in both human and animal pathogens because therapeutic-resistant infections may lead to longer hospitalization, longer treatment time, failure of treatment therapy, and the need for treatment with more toxic or costly antibiotics, as well as an increased likelihood of death.

A vast amount of work has been undertaken to understand the contribution and roles played by hospital and community settings in the dissemination and maintenance of ARB infections in humans. A particular area of focus in terms of exposure in a community setting has been antibiotic use in livestock production and the presence of eARB and pARB in food of animal origin. In 2011, the Codex Alimentarius Commission [established in 1963 by the Food and Agriculture Organization of the United Nations (FAO) and the World Health Organization (WHO) to harmonize international food standards, guidelines, and codes of practice to protect the health of consumers and ensure fair trade practices in the food trade] released guidelines on processes and methodologies for applying risk analysis methods to foodborne antimicrobial resistance related to the use of antimicrobials in veterinary medicine and agriculture (Codex Alimentarius Commission 2011).

Other sources of antibiotics and other antimicrobials in the environment are human sewage (Dolejska et al. 2011), intensive animal husbandry, and waste from the manufacture of pharmaceuticals (Larsson et al. 2007). The environmental consequences from the use and release of antibiotics from various sources (Kümmerer 2009a, 2009b) and the HGT of antibiotic-resistance genes (ARG) between indigenous environmental and pathogenic bacteria and their resistance determinants (Börjesson et al. 2009; Chagas et al. 2011; Chen et al. 2011; Cummings et al. 2011; Forsberg et al. 2012; Gao et al. 2012; Qiu et al. 2012) has yet to be quantified, but is of global concern (Finley et al. 2013; WHO 2012a). The genetic elements encoding for the ability of microorganisms to withstand the effects of an antimicrobial agent are located either chromosomally or extrachromosomally and may be associated with mobile genetic elements such as plasmids, integrons, gene cassettes, or transposons, thereby enabling horizontal and vertical transmission from resistant to previously susceptible strains. From an HHRA point of view, the emergence of ARB in source and drinking water (De Boeck et al. 2012; Isozumi et al. 2012; Shi et al. 2013) further highlights the need to place these emerging environmental risks in perspective. Yet, assessing the range of environmental contributions to antibiotic resistance may not only be complicated by lack of quantitative data but also by the need to coordinate efforts across different agencies that may have jurisdiction over environmental risks versus human and animal health.

A key consideration for ARB development in the environment is that resistance genes can be present due to natural occurrence (D’Costa et al. 2011). Further, the use of antimicrobials in crops, animals, and humans provides a continued entry of antibiotics to the environment, along with possible novel genes and ARB. A summary of the fate, transport, and persistence of antibiotics and resistance genes after land application of waste from food animals that received antibiotics or following outflow to surface water from sewage treatment has emphasized the need to better understand the environmental mechanisms of genetic selection and gene acquisition as well as the dynamics of resistance genes (resistome) and their bacterial hosts (Chee-Sanford et al. 2009; Crtryn 2013). For example, the presence of antibiotic residues in water from pharmaceutical manufacturers in certain parts of the world (Fick et al. 2009), ponds receiving intensive animal wastes (Barkovskii et al. 2012), aquaculture waters (Shah et al. 2012), and sewage outfalls (Dolejska et al. 2011) are important sources, among others, leading to the presence of ARG in surface waters. In particular, the comparatively high concentrations of antibiotics found in the effluent of pharmaceutical production plants have been associated with an increased presence of ARG in surface waters (Kristiansson et al. 2011; Li et al. 2009, 2010). Most recently, 100% sequence identity of ARG from a diverse set of clinical pathogens and common soil bacteria (Forsberg et al. 2012) has highlighted the potential for environmental HGT between eARB and pARB.

Despite these concerns, few risk assessments have evaluated the combined impacts of antibiotics, ARG, and ARB in the environment on human and animal health (Keen and Montforts 2012). Recent epidemiological studies have included evaluation of ARB in drinking water and the susceptibility of commensal *Escherichia coli* in household members. For example, Coleman et al. (2012) reported that water, along with other factors not directly related to the local environment, accounted for the presence of resistant *E. coli* in humans. In many studies, native bacteria in drinking water systems have been shown to accumulate ARG (Vaz-Moreira et al. 2011).

In addition to addressing environmental risks arising from the development of antibiotic resistance, we should also consider the low probability but high impact “one-time-event” type of risk. This exceedingly rare event that results in the transfer of a novel (to clinically important bacteria) resistance gene from a harmless environmental bacterium to a pathogen need happen only once if a human is the recipient of the novel pARB. Unlike the emergence of SARS (severe acute respiratory syndrome) and similar viruses where, in hindsight, the risk factors are now well understood (Swift et al. 2007), the conditions for a “one-time event” could occur in a range of “normal” habitats. Once developed, the resistant bacterium/gene has a possibility to spread between humans around the world [such as seen with the spread of NDM-1 (New Delhi metallo-beta-lactamase-1) resistance (Wilson and Chen 2012)], promoted by our use of antibiotics. Although it seems very difficult to quantify the probability for such a rare event (including assessing the probability for where it will happen and when), there is considerable value in trying to identify the risk factors (such as pointing out critical environments for HGT to occur, or identifying pharmaceutical exposure levels that could cause selection pressures and hence increase the abundance of a given gene). After such a critical HGT event, we may then move into a more quantitative kind of HHRA.

The overall goal of the workshop (Antimicrobial Resistance in the Environment: Assessing and Managing Effects of Anthropogenic Activities) was to identify the significance of ARB within the environment and to map out some of the complexities involved in order to identify research gaps and provide statements on the level of scientific understanding of various ARB issues. A broad range of international delegates, including academics, government regulators, industry members, and clinicians, discussed various issues. The focus of this review arose from discussions of improving our understanding of human health risks—in addition to epidemiological studies—by developing HHRAs to explore potential risks and inform risk management. Because the end goal of an assessment depends on the context (e.g., research, regulation), we provide a generic approach to undertaking an HHRA of environmental ARB that can be adapted to the users’ interest (conceptualized in Figure 1). Given the many uncertainties, we also highlight identified research gaps.

## General Considerations for an Assessment of Environmental ARB Risks

Understanding other ongoing relevant international activities and the types of antibiotics used provide good starting points to aid in framing a risk assessment of ARB. The Codex Alimentarius Commission (2011) described eight principles that are specific to risk analysis for foodborne antimicrobial resistance, several of which are generally applicable to a HHRA of environmental ARB. Examples include the recommendations of the *Joint FAO/WHO/OIE Expert Meeting on Critically Important Antimicrobials* (Food and Agriculture Organization of the United Nations/World Health Organization/World Organisation for Animal Health 2008) and the WHO Advisory Group on Integrated Surveillance of Antimicrobial Resistance (WHO 2012b), which provided information for setting the priority antibiotics for a human risk assessment. It should be noted that there are significant national and regional differences in antibiotic use, resistance patterns, and human exposure pathways.

In general, risk assessments are framed by identifying risks and management goals, so the assessment informs the need for possible management options and enables evaluation of management success. The consensus of workshop participants was that management could best be applied at points of antibiotic manufacturing and use, agricultural operations including aquaculture, and wastewater treatment plants (Pruden et al. 2013). Assessing the relative impact of managing any particular part of a system is hampered by the lack of knowledge on the relative importance of each part of the system for the overall risk. That is, as recently stated by the WHO (2013), “AMR is a complex problem driven by many interconnected factors so single, isolated interventions have little impact and coordinated actions are required.” Hence, a starting point for an assessment of environmental antibiotic-resistance risks intended to aid risk management is a theoretical risk assessment pathway based on *a*) local surveillance data on the occurrence and types of antibiotics used in human medicine, crop production, animal husbandry, and companion animals; *b*) information on ARG and ARB in the various environmental compartments (in particular, soil and aquatic systems including drinking water); and *c*) related disease information. This assessment should be amended by discussion with the relevant stakeholders, which requires extensive risk communication and could form part of the multicriteria decision analysis (MCDA) approach discussed in detail below. As a result of the workshop, Pruden et al. (2013) also advocate coupling environmental management and mitigation plans with targeted surveillance and monitoring efforts in order to judge the relative impact and success of the interventions.

To undertake a useful human health risk assessment, some details require quantitative measures. Thus, the key issue is how experimental and modeling approaches can be used to derive estimates. Furthermore, hazard concentration, time, and environmental compartment-dependent aspects should also be taken into account. First, the current understanding is that for non-mutation-derived antibiotic resistance to develop in environmental bacteria (including pathogens that may actively grow outside of hosts) to develop into eARB/pARB (Figure 1, processes 1 and 2), a selective pressure (i.e., presence of antibiotics or antibiotic-resistance determinants) must be maintained over time in the presence of ARG; for existing pARB released into the environment, survival in environmental media is the critical factor. However, the exact mechanisms and quantitative relationships between selective pressures and ARB development have yet to be elucidated, and they may be different depending on the antibiotic, bacterial species, and resistance mechanisms involved. In cases where selective pressure is removed, the abundance of antibiotic-resistance ARB may be reduced, but not to extinction. (Andersson and Hughes 2010, 2011; Cottell et al. 2012). Even a small number of ARB at the community level represents a reservoir of ARG for horizontal transfer once pressure is reapplied. Because it seems inevitable that ARB will eventually develop against any antibiotic (Levy and Marshall 2004), the key management aim seems to be to delay and confine such a development as much as possible.

Second, a robust quantitative risk assessment will require rates of HGT and/or gene mutations in the relevant compartments (Figure 1, processes 3–5) to be described for different combinations of donating eARB strains and receiving pARB strains. The lack of quantitative estimates for mutation/HGT of ARG is a major data gap.

Third, traditional microbial risk assessment dose–response approaches (Figure 1, processes 6 and 8) could be used to address the likelihood of infection [Codex Alimentarius Commission 2011; U.S. EPA and U.S. Department of Agriculture/Food Safety and Inspection Service (USDA/FSIS) 2012], but the novel aspect required here—in addition to HGT and ARB selection—would be to address quantitative dose–response relationships for eARB (in the presence of a sensitive pathogen in or on a human) (Figure 1, processes 3 and 6). Importantly, the key difference from traditional HHRA undertaken in some jurisdictions is that it is essential to include environmental processes to fully assess human risks associated with antibiotic resistance.

Therefore, the type of information that should be documented for a human health–oriented risk assessment of environmental ARB includes the following [adapted from Codex Alimentarius Commission (2011)]:

Clinical and environmental surveillance programs for antibiotics, ARB, and their determinants, with a focus on regional data reporting the types and use of antibiotics in human medicine, crops, and commercial and companion animals, as well as globally where crops and food animals are produced
Epidemiological investigations of outbreaks and sporadic cases associated with ARB, including clinical studies on the occurrence, frequency, and severity of ARB infections
Identification of the selection pressures (time and dose of selecting/coselecting agents) required to select for resistance in different environments, and subsequent HGT to human-relevant bacteria, both based on reports describing the frequency of HGT and uptake of ARG into environmental bacteria, including environmental pathogens, in previously identified hot spots
Human, laboratory, and/or field animal/crop trials addressing the link between antibiotic use and resistance (particularly regional data)
Investigations of the characteristics of ARB and their determinants (*ex situ* and *in situ*)
Studies on the link between resistance, virulence, and/or ecological fitness (e.g., survivability or adaptability) of ARB
Studies on the environmental fate of antibiotic residues in water and soil and their bioavailability associated with the selection of ARB in any given environmental compartment, animal, or human host resulting in pARB
Existing risk assessments of ARB and related pathogens.

In summary, many sources of data are required to undertake a human health risk assessment for environmental ARB, and much of the data may be severely limited (particularly for a quantitative assessment). Thus, the final risk assessment report should emphasize the importance of the evidence trail and weight of evidence for each finding. Furthermore, when models are constructed, previously unused data sets should be considered for model verifications where possible.

## Applicability of Traditional Risk Assessment Approaches

Human health risk assessment of antibiotics in the environment builds on traditional chemical risk assessments (National Research Council 1983), starting, for example, with an acceptable daily intake (ADI) based on resistance data (VICH Steering Committee 2012). A corresponding metric for environmental antibiotic concentration could be developed based on the concept of the minimum selective concentration (MSC) (Gullberg et al. 2011), defined as the minimum concentration of an antibiotic agent that selects for resistance. Unlike the traditional chemical risk assessment approach, with the MSC assay it would be necessary to address the human health effects arising from ARGs and the resistance determinants that give rise to ARB, including resistance associated with mutations (Figure 1, processes 1 and 2). In the absence of specific data, an MSC assay could inform a risk assessor of the selective concentration of a pharmaceutical or complex mixture of compounds in a matrix of choice, allowing description of thresholds for significant ARB development.

Pathogen risks may be evaluated through microbial risk assessment (MRA), a structured, systematic, science-based approach that builds on the chemical risk assessment paradigm; the MRA involves *a*) problem formulation (describing the hazards, risk setting, and pathways), *b*) exposure assessment of the hazard (ARB, ARG), *c*) dose–response assessment that quantifies the relationship between hazard dose and pARB infection in humans (Figure 1, processes 6 and 7), and *d*) combination of these procedures to characterize risk for the various pathways of exposure to pathogens identified to be assessed. An MRA is used qualitatively or quantitatively to evaluate the level of exposure and subsequent risk to human health from microbiological hazards. In the context of antibiotic-resistant microorganisms, environmental MRA is in its infancy but is needed to address resistant bacteria and/or their determinants. The MRA was originally developed for fecal pathogen hazards in food and water [ILSI (International Life Sciences Institute) 1996], with more recent modifications to include biofilm-associated environmental pathogens such as *Legionella pneumophila* (Schoen and Ashbolt 2011). Some human pathogens can grow in the environment (and may become pARB; Figure 1, processes 1 and 2), and many will infect only compromised individuals (generally termed opportunistic pathogens).

Over the past 20 years, the MRA has largely evolved by input from the international food safety community, and it is now a well-recognized and accepted approach for food safety risk analysis. In 1999, the Codex Alimentarius adopted the *Principles and Guidelines for the Conduct of Microbiological Risk Assessment* (CAC/GL-30) (Codex Alimentarius Commission 2009). The most recent Codex Alimentarius guidelines for risk analysis of foodborne antimicrobial resistance include eight principles (Codex Alimentarius Commission 2011), and in the United States, MRA guidelines for food and water (U.S. EPA and USDA/FSIS 2012) continue to use the four-step framework originally described for chemical risk assessment. Several ARB risk assessments have been published and reviewed in recent years (Geenen et al. 2010; McEwen 2012; Snary et al. 2004). However, nearly all of these studies focus on foodborne transmission; human health risk assessments dealing with ARB transmission via various environmental routes or direct contact with ARG are sparse.

For example, Geenen et al. (2010) studied extended-spectrum beta-lactamase (ESBL)-producing bacteria and identified the following risk factors: previous admission to health-care facilities, use of antimicrobial drugs, travel to high-endemic countries, and having ESBL-positive family members. The authors concluded that an environmental risk assessment would be helpful in addressing the problem of ESBL-producing bacteria but that none had been performed.

*Hazard identification and hazard characterization.* Unfortunately, we are unaware of data that quantitatively link ARG uptake and human health effects (Figure 1, processes 3 and 6). What data do exist and are rapidly improving in quality, however, are on the presence of ARGs within various environmental compartments (Allen et al. 2009; Cummings et al. 2011; Ham et al. 2012), specifically on clinically relevant resistance genes within soils (Forsberg et al. 2012) (Figure 1, process 1). Precursors that lead to the development of ARB hazards include ARG and mechanisms to mobilize these genes, antibiotics, and coselecting agents (Qiu et al. 2012; Taylor et al. 2011) along with gene mutations (Gillings and Stokes 2012). Depending on the presence of recipient bacteria, these processes generate either eARB or pARB (Figure 1, processes 1 and 2).

In regard to the numerous parameters relevant to individual environmental compartments, we are not aware of the availability of comprehensive data on *a*) antibiotic resistance development by antibiotics and other coselecting agents; *b*) the flow of ARG (resistome) and acquisition elements (e.g, integrons) in native environmental compartment bacteria; or *c*) the likely range in rates of horizontal and vertical gene transfer within environmental compartments. Nonetheless, factors that are considered important include the range of potential pathways involving the release of antibiotics, ARG, and ARB into and amplifying in environmental compartments such as the rhizosphere, bulk soil, compost, biofilms, wastewater lagoons, rivers, sediments, aquaculture, plants, birds, and wildlife.

With respect to antibiotics, in general, the following information is required to aid hazard characterization: *a*) a list of the local antibiotic classes of concern, *b*) what is known of their environmental fate, and *c*) where they may accumulate, in particular, environmental compartments (e.g., the rhizosphere, general soil, compost, biofilms, wastewater lagoons, rivers, sediments, aquaculture, plants, birds, wildlife, farm animals, or companion animals). Selection for ARB (Figure 1, process 2) will depend on the type and *in situ* bioavailability of selecting/coselecting agents, the abundance of bacterial host, and the abundance of AR determinants.

Selection for ARB is further modulated by the nutritional status of members of the relevant bacterial community because high metabolic activity and high cell density promote bacterial community succession and HGT (Brandt et al. 2009; Sørensen et al. 2005). In contrast, HGT is relatively independent of antibiotics—although antibiotics and ARB may be co-transported (Chen et al. 2013)—and increases in HGT rates are thought to occur in stressed bacteria. For example, integrase expression can be up-regulated (increased) by the bacterial SOS response (process for DNA repair) in the presence of certain antibiotics (Guerin et al. 2009).

Although quantitative data that describe the development of pARB in the environment are lacking, ample evidence exists for the co-uptake by an antibiotic-sensitive pathogen in the presence of an antibiotic, ARG (such as on a plasmid with metal resistance), and/or carbon utilization genes (Knapp et al. 2011; Laverde Gomez et al. 2011), or as demonstrated *in vitro* for a disinfectant/nanomaterial (Qiu et al. 2012; Soumet et al. 2012).

The spatial distribution of organisms (opportunity for close proximity) may also affect gene transfer, which results from inherent patchiness, soil structure, presence of substrates, and so forth. In considering gene transfer rates, there may be hot spots of activity; for example, there is evidence for HGT of clinically relevant resistance genes between bacteria in manure-impacted soils (Forsberg et al. 2012) and in association with the rhizosphere because of its organic-rich conditions (Pontiroli et al. 2009). In addition, selection pressures for subsequent proliferation of eARB may be higher in these hot spot environments (Brandt et al. 2009; Li et al. 2013). Therefore, it is important to recognize likely zones of high activity during the problem formulation and hazard characterization stages of a risk assessment, and when using sampling to identify *in situ* exchange rates. As an example marker of anthropogenic impact with potential to predict the impact on the mobile resistome, class 1 integrons could be used because of their ability to integrate gene cassettes that confer a wide range of antibiotic and biocide resistance (Gaze et al. 2011). In semi-pristine soils, prevalence may be two or three orders of magnitude lower than in impacted soils and sediments (0.001 vs. 1%, respectively) (Gaze et al. 2011; Zhu et al. 2013).

In addition to a huge diversity of eARB hazards, there are several pathogens that could be evaluated in microbial risk assessments: *a*) foodborne and waterborne fecal pathogens represented by *Campylobacter jejuni*, *Salmonella enterica*, or various pathogenic *E. coli*; and *b*) environmental pathogens, such as respiratory, skin, or wound pathogens represented by *Legionella pneumophila, Staphylococcus aureus*, and *Pseudomonas aeruginosa.* Each of these fecal and environmental pathogens is well known to be able to acquire ARG; thus, given further data on environmental HGT rates, they could be used as reference pathogens in microbial risk assessments. However, what is much more problematic for risk assessment—and a current limiting factor—is the rate at which the indigenous bacteria transfer resistance to these pathogens within each environmental compartment and within the human/animal host (Figure 1, processes 3–5). Methods to model and experimentally derive relevant information on these environmental issues are discussed below in “Environmental Exposure Assessment.” Data on HGT within the human gastrointestinal tract have been summarized by Hunter et al. (2008).

*Dose–response relationships.* To properly characterize human risks, it is typical to select hazards for which there are dose–response health data described either deterministically or stochastically, as available for the reference enteric pathogens (e.g., *Campylobacter jejuni*, *Salmonella enterica*, *E. coli*) (Schoen and Ashbolt 2010), but these dose–response health data have yet to be quantified for the skin/wound reference pathogens (Mena and Gerba 2009; Rose and Haas 1999). However, as noted above for processes 1–5, (Figure 1), an important difference for ARB is the need to account for the phenomena associated with selective environmental pressures for the development of ARB, and ultimately that form the human infective dose of either eARB or pARB. The exact mechanisms and dose–response relationships have yet to be elucidated, and may be different depending on the bacterial species and resistance mechanisms involved. Nevertheless, it seems reasonable for the noncompromised human exposed to a pARB to fit the published dose–response/infection relationship (e.g., derived from “feeding” trials with healthy adults or from information collected during outbreak investigations) for strains of the same pathogen without antibiotic resistance. What appears more limiting are dose–response models that describe the probability of illness based on the conditional probability of infection and including people who are already compromised, such as those undergoing antibiotic therapy. Although there is definitive data on pARB being more pathogenic or causing more severe illness than their antimicrobial-susceptible equivalents (Barza 2002; Helms et al. 2004, 2005; Travers and Barza 2002), that may not always be the case (Evans et al. 2009; Wassenaar et al. 2007). Clear examples of excess mortality include associated blood stream infections for methicillin-resistant *Staphylococcus aureus* (MRSA) and from third generation cephalosporin-resistant *E. coli* (G3CREC). In 2007 in participating European countries, 27,711 cases of MRSA were associated with 5,503 excess deaths and 255,683 excess hospital days, and 15,183 episodes of G3CREC blood stream infections were responsible for 2,712 excess deaths and 120,065 extra hospital days (de Kraker et al. 2011). The authors predicted that the combined burden of resistance of MRSA and G3CREC will likely lead to a predicted incidence of 3.3 associated deaths per 100,000 inhabitants in 2015. Yet for many regions of the world, such predictions are less well understood.

The final step of MRA is risk characterization, which integrates the outputs from the hazard identification, the hazard characterization, dose response, and the exposure assessment with the intent to generate an overall estimate of the risk. This estimate may be expressed in various measures of risk, for example, in terms of individual or population risk, or an estimate of annual risk based on exposure to specific hazard(s). Depending on the purpose of the risk assessment, the risk characterization can also include the key scientific assumptions used in the risk assessment, sources of variability and uncertainty, and a scientific evaluation of risk management options.

## Environmental Exposure Assessment

Based on our conceptualization of the processes important to undertake HHRA of ARB (Figure 1), most elements related to ARB development in environmental media (processes 1, 2, and 4) have been addressed above in “Hazard identification and hazard characterization.” Here we focus on describing important environmental compartments for and human exposure to ARB (Figure 1, processes 3 and 6). Concentrations of environmental factors (such as antibiotics) and ARB, along with their fate and transport to points of human uptake, are critical to exposure assessment. For a particular human health risk assessment of ARB, it would be important to select/expand on individual pathway scenarios (identifying critical environmental compartments to human contact) relevant to the antibiotic/resistance determinants identified in the problem formulation and hazard characterization stages.

Compartments of potential concern include soil environments receiving animal manure or biosolids, compost, and lagoons, rivers, and their sediments receiving wastewaters (Chen et al. 2013). More traditional routes of human exposures to contaminants that could include eARB and pARB are drinking water, recreational and irrigation waters impacted by sewage and/or antibiotic production wastewaters, food, and air affected by farm buildings and exposure to farm animal manures, as discussed by Pruden et al. (2013). What is emerging as an important research gap is the *in situ* development of ARB within biofilms (Boehm et al. 2009) and their associated free-living protozoa that may protect and transport ARB (Abraham 2010) to and within drinking water systems (Schwartz et al. 2003; Silva et al. 2008). This latter route could be particularly problematic for hospital drinking water systems where an already vulnerable population is exposed. In addition, with the increasing use and exposure to domestically collected rainwater, atmospheric fallout of ARB may “seed” household systems (Kaushik et al. 2012).

After identifying antibiotic concentrations and pathogen densities in the environment, as well as possible levels and rates of ARB generation in each environmental compartment, a range of fate and transport models are available to estimate the amounts of antibiotics, pathogens, ARB, and ARG at points of human contact (Figure 1, processes 3 and 6). Such models are largely based on hydrodynamics, with pathogen-specific parameters to account for likely inactivation/predation in soil and aquatic environments, such as sunlight inactivation (Bradford et al. 2013; Cho et al. 2012; Ferguson et al. 2010). A key aspect of the fate and transport models is the ability to account for the inherent variability of any system component. In addition, our uncertainties in assessing model parameter values should be factored into fate and transport models such as by using Bayesian synthesis methods (Albert et al. 2008; Williams et al. 2011). To better account for parameter uncertainties, more recent models are including Bayesian learning algorithms that help to integrate information using meteorologic, hydrologic, and microbial explanatory variables (Dotto et al. 2012; Motamarri and Boccelli 2012). Overall, these models also help to identify management opportunities to mitigate exposures to ARB and antibiotics and are an important aspect in describing the pathways of hazards to points of human exposure in any risk assessment.

## MCDA and Risk Ranking Methods

Considering the complexity of exposure pathways associated with environmental ARB risks and the large uncertainty in the input data for some nodes along the various exposure pathways, outputs would inevitably be difficult for decision makers to interpret and could in fact be counterproductive. Thus, there is merit in considering decision analysis approaches to prioritize risks, guide resource allocation and data collection activities, and facilitate decision making. Although there is a range of ranking options, uses of weightings, and selection criteria (Cooper et al. 2008; Pires and Hald 2010), as well as failure mode and effects analysis (Pillay and Wang 2003), in the overall area of microbial risk assessment, there is a consolidation to MCDA approaches that may include Bayesian algorithms (Lienert et al. 2011; Ludwig et al. 2013; Ruzante et al. 2010).

Approaches such as MCDA are designed to provide a structured framework for making choices where multiple factors need to be considered in the decision-making process. MCDA is a well-established tool that can be used for evaluating and documenting the importance assigned to different factors in ranking risks (Lienert et al. 2011), albeit heavily dependent on expert opinion. In the context of MRA, MCDA has been used to rank foodborne microbial risks based on multiple factors, including public health, market impacts, consumer perception and acceptance, and social sensitivity (Ruzante et al. 2010), as well as to prioritize and select interventions to reduce pathogen exposures (Fazil et al. 2008). Examples of MCDA applications in structuring decisions for managing ecotoxicological risks have also been reported (Linkov et al. 2006; Semenzin et al. 2008) and provide useful MCDA approaches. MCDA could be used, for example, to evaluate and rank the relative risks between habitats highly polluted with antibiotics, ARG, and ARG determinants, as described above for possible hot spots for HGT and development of ARB. MCDA could be applied to evaluate the relative contribution of coselecting agents (e.g., detergents, biocides, metals, nanomaterials) from various sources to the overall risk of ARB in the environment. Moreover, for a range of antibiotics considered to be of environmental concern, MCDA approaches could be used for risk ranking according to criteria based on relevant contributing factors (e.g., mobility of resistance determinants in genetic elements, antibiotic-resistance transfer rates in different environmental compartments, accumulation levels of antibiotics in environmental compartments, environmental fate and transport to exposure points). In the MCDA process, it is also important to identify low probability but high impact “one-time-event” types of risk.

Because MCDA techniques rely on expert opinion (which is often regarded as a limitation of such approaches), well-structured and recognized elicitation practices should be used in order to avoid introduction of biases and errors by subjective scoring. In contrast, one of the main advantages of MCDA techniques is that they capture a consensus opinion among an expert group about the most relevant criteria and their relative weight on the decision.

## Important Research Gaps Affecting Progress of HHRA of Antibiotic Resistance

There are several research gaps that need to be addressed. In particular, specific attention should be paid to contaminated habitats (hot spots) in which antibiotics, coselecting agents, bacteria carrying resistance determinants on mobile genetic elements, and favorable conditions for bacterial growth and activity—all conditions expected to favor HGT—prevail at the same time. However, because these data are currently very limited, workshop participants evaluated alternative ways and possible experimental methods to address these data gaps for HHRA as they relate to the processes identified in Figure 1.

*Assays to determine MSC (processes 1, 2, and 4).* Assays could be developed to measure MSC (Gullberg et al. 2011) for a range of antibiotics and environmental conditions. For example, assays could be developed and validated in sandy and clay soils, different sediments, and water types, with isogenic pairs of the model organism inoculated into the matrix of choice and subjected to a titration of the selective agent to sufficiently high dilution. Selection at subinhibitory concentrations and assay development for environmental matrices are key areas of research that need to be addressed. However, overall care is needed when interpreting *ex situ* studies and extrapolating to *in situ* environmental conditions, as well as in dealing with ill-defined hazard mixtures in the environment.

*Assays to identify environmental hot spots (processes 1, 2, and 4).* Hot spots, locations where a high-level of HGT and antibiotic resistance develop, may, for instance, include aquatic environments affected by pharmaceutical industry effluents, aquaculture, or sewage discharges, as well as terrestrial environments affected by the deposition of biosolids or animal manures. The degree of persistence of antibiotic resistance (i.e., the rate by which resistance disappears without having an environmental selection pressure for resistance) must also be considered for risk assessment and will depend on the fitness cost of resistance. However, the fitness costs within complex and variable environments are difficult to assess. Furthermore, standard methods have not been developed for evaluating environmental selection pressures in complex microbial communities, but several experimental approaches are possible and have been described elsewhere (Berg et al. 2010; Brandt et al. 2009).

The approaches identified by Berg et al. (2010) and Brandt et al. (2009) could be laboratory based (to assess the potency of known compounds/mixtures) or applied in the field to assess whether the environment in question (with, for example, its unknown mixture of chemicals) is a hot spot. Defining “critical exposure levels” is therefore an important HHRA output to aid management activities, which will likely vary between and within environmental compartments, depending on the location.

*Screening for novel resistance determinants (to reduce process 2).* Screening procedures could be introduced early in the development cycle of novel antibiotics to ensure that existing resistance determinants are not prevalent in environmental compartments. Marked recipient strains could be inoculated into environmental matrices [e.g., soil, biosolids, or fecal slurry (with sterilized matrix equivalents as negative controls)], incubated, and then seeded onto media containing the study compound along with a selective antibiotic to recover marked recipient strains demonstrating resistance. Plasmids, or the entire genome of the recipient, could then be cloned into small insert expression vectors, transformed into *E. coli* or other hosts, and seeded back onto media containing the study compound. In this way, novel resistance determinants would be identified.

Alternatively, functional metagenomics could be used to identify novel resistance determinants in metagenomic DNA (Allen et al. 2009). In brief, DNA would be extracted from an environmental sample, cloned into an expression vector, and transformed into a bacterial host such as *E. coli*. Transformants could then be screened on the study compound and resistance genes identified using transposon mutagenesis followed by sequencing and bioinformatic analyses. This would allow detection of novel resistance determinants that may not be plasmid borne but may transfer to other pathogens.

*Dose–response data needs (processes 3, 5, and 6).* We were unaware of dose–response data representing a combined ARG and a recipient, previously susceptible pathogen dose, and human or animal disease (Figure 1, processes 3 and 5). In contrast, various examples illustrate increased morbidity and mortality when humans are exposed to pARB, as described above in “Dose–response relationships.” Hence, existing published dose–response models for nonresistant pathogens (Haas et al. 1999) may not be appropriate to use beyond the end point of infection, and further dose–response models that address people of various life-stages need to be described and summarized to facilitate pARB risk assessments. There is also a need to develop dose–response information for secondary illness end points (sequelae) resulting from pARB infections.

Regarding antibiotic concentration and time of exposure giving rise to selection of pARB within a human (for co-uptake of eARB and a sensitive pathogen), safety could be based on the effective concentration for the specific antibiotic under consideration. In other words, screening values to determine whether further action is warranted could be derived from the acute or mean daily antibiotic intake, with uncertainty factors applied as appropriate, until future research is undertaken on pathogen antibiotic-response changes in the presence of specific antibiotic treatment. Alternatively, epidemiological data from existing clones of antibiotic-resistant strains (e.g., NDM-1) could provide useful data for dose–response and exposure assessments.

*Options for ranking risks (overall HHRA).* In the absence of fully quantitative data to undertake a HHRA, risk-ranking approaches based on exposure assessment modeling could be adopted and developed to inform allocation of resources for data generation as part of an HHRA of ARB. Evers et al. (2008) presented one such approach in the context of food safety for estimating the relative contribution of *Campylobacter* spp. sources and transmission routes on exposure per person-day in the Netherlands. Their study included 31 transmission routes related to direct contact with animals and ingestion of food and water, and resulted in a ranking of the most significant sources of exposure. Although their study focused on foodborne transmission routes and did not deal with antibiotic-resistant *Campylobacter* strains, a similar approach could be applied to estimate human exposure to ARB hazards using the environmental exposure pathways described by Evers et al. (2008). This would require data on the prevalence of ARG and ARB, as well as levels of antibiotics present in all exposure routes to be considered in the risk assessment. Although such an approach is probably not currently feasible, improved environmental data for a select number of pathogen–gene combinations could be developed in the future.

An alternative approach to capturing knowledge of experts and other stakeholders could be to develop a Bayesian network based on expert knowledge and add to that as data become available, as described for campylobacters in foods by Albert et al. (2008).

## Conclusions

Because we are addressing an international problem and because the precautionary approach is used in many jurisdictions, there are many risk management approaches that can be implemented now, before antibiotic-resistance issues worsen, as noted in the related risk management paper resulting from the workshop (Pruden et al. 2013). Furthermore, many current risk management schemes start the process from a management perspective and delve into quantitative assessments as needed in order to improve risk management actions, such as in the WHO water safety plans (WHO 2009). We propose that environmental aspects of antibiotic-resistance development be included in the processes of any HHRA addressing ARB. In general terms, an MRA appears suitable to address environmental human health risks posed by the environmental release of antibiotics, ARB, and ARG; however, at present, there are still too many data gaps to realize that goal. Further development of this type of approach requires data mining from previous epidemiological studies to aid in model development, parameterization, and validation, as well as in the collection of new information, particularly that related to conditions and rates of ARB development in various hot spot environments, and for various human health dose–response unknowns identified in this review. In the near-term, options likely to provide a first-pass assessment of risks seem likely to be based on MCDA approaches, which could be facilitated by Bayesian network models. Once these MRA models gain more acceptance, they may facilitate scenario testing to determine which control points may be most effective in reducing risks and which risk-driving attributes should be specifically considered and minimized during the development of novel antibiotics.
